# Nanofibril Alignment during Assembly Revealed by an X-ray Scattering-Based Digital Twin

**DOI:** 10.1021/acsnano.1c07769

**Published:** 2022-02-01

**Authors:** V. Krishne Gowda, Tomas Rosén, Stephan V. Roth, L. Daniel Söderberg, Fredrik Lundell

**Affiliations:** † Department of Engineering Mechanics, Royal Institute of Technology, 100 44 Stockholm, Sweden; ‡ FLOW, Royal Institute of Technology, 100 44 Stockholm, Sweden; ¶ Treesearch, Royal Institute of Technology, 100 44 Stockholm, Sweden; § Wallenberg Wood Science Center, Royal Institute of Technology, 100 44 Stockholm, Sweden; ∥ Department of Fibre and Polymer Technology, Royal Institute of Technology, 100 44 Stockholm, Sweden; ⊥ Deutches Elektronen-Synchrotron DESY, 22607 Hamburg, Germany

**Keywords:** alignment, cellulose nanofibrils, flow-focusing, X-ray scattering, rotary diffusion, assembly

## Abstract

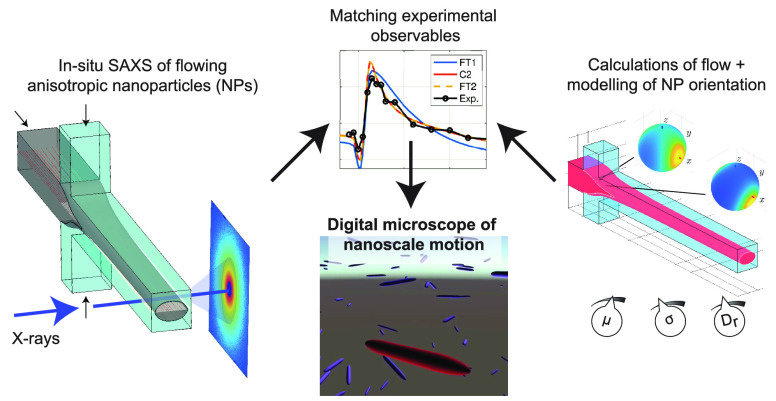

The nanostructure, primarily particle orientation, controls mechanical and functional (e.g., mouthfeel, cell compatibility, optical, morphing) properties when macroscopic materials are assembled from nanofibrils. Understanding and controlling the nanostructure is therefore an important key for the continued development of nanotechnology. We merge recent developments in the assembly of biological nanofibrils, X-ray diffraction orientation measurements, and computational fluid dynamics of complex flows. The result is a digital twin, which reveals the complete particle orientation in complex and transient flow situations, in particular the local alignment and spatial variation of the orientation distributions of different length fractions, both along the process and over a specific cross section. The methodology forms a necessary foundation for analysis and optimization of assembly involving anisotropic particles. Furthermore, it provides a bridge between advanced in operandi measurements of nanostructures and phenomena such as transitions between liquid crystal states and in silico studies of particle interactions and agglomeration.

## Introduction

The coupling between nonspherical particles or long molecules suspended in a liquid and the liquid itself is critical when preparing advanced materials from high-aspect-ratio nanofibrils, which have a width of typically a few nanometers and are abundant in the biological world. A few examples are macromolecular building blocks such as protein- and cellulose nanofibrils (PNFs and CNFs) and viruses.^1−5^ From a sustainability perspective, cellulose nanofibrils are of particular interest, since they have the potential to contribute to the solution of, e.g., plastic pollution.^6^ There are also a wide range of human-made nonspherical nanoparticles that are used to prepare nanostructured anisotropic materials, e.g., nanotubes, nanowires, and nanoflakes.^7,8^

Measuring, understanding, predicting, manipulating, and utilizing fibril alignment in complex and transient flow situations used for assembly remain challenging on the nanoscale^9^ as well as in a wide range of other situations such as tomato fibers in ketchup processing,^10^ crystal aggregates in lava flow,^11^ and paper fibers in papermaking.^12^

In the absence of external fields or interfaces, the alignment (or actually the orientation distribution) of nonspherical particles will be the integrated result of three main mechanisms:^13^ (i) velocity gradients causing particle rotation, (ii) Brownian diffusion, and (iii) particle interactions through direct contact, electrostatics and/or hydrodynamics. A key parameter controlling the alignment behavior is the Peclet number *Pe*, defined as the ratio between the velocity gradients (or rate of deformation) and the Brownian diffusion. For *Pe* ≪ 1, diffusion dominates and the flow cannot align particles, while for *Pe* ≫ 1, diffusion is insignificant and the flow controls the alignment completely. Concerning (i), the flow around and the hydrodynamic forces exerted on an ellipsoidal particle in an infinite linearly varying flow was solved by Jeffery (1922).^14^ Modeling of (ii) and (iii) have turned out to be more complex. A wide range of analytical, experimental, and numerical models are available to elucidate the three mechanisms. Such models can incorporate various aspects, such as particle elasticity, morphology, concentration, and surface charges.^13,15^

In this work, we will use data from X-ray scattering measurements of fibril alignment to calibrate alignment models in situ. Furthermore, one calibrated model is used to understand the details of fibril alignment in complex flows used for filament assembly. In this context, additional challenges present themselves. It is, in fact, nontrivial to reconstruct the complete three-dimensional orientation distribution of CNFs in flow from scattering data; recent progress demonstrate that scattering from multiple directions is necessary to reconstruct the orientation state correctly.^16^ In complex flows where the orientation state varies in space, this quickly becomes overwhelming. Tomographic methods could be considered, but the requirements in terms of experiment design and measurement time needed make them hard to use for in situ measurements in complex flows or extensive parameter variations.^17^

Here, another approach is used when calibrating the models. We reconstruct the actually measured experimental result from simulated orientation distributions. The difference between the experimental and simulated “measurements” is then used to calibrate the simulation model. We thus fill the gap between (i) our previous work on modeling of the flow^18^ and (ii) creation of projected orientation distributions from simulations of collective particle dynamics.^19,20^ The result is a digital twin concept that combines experimental and numerical methods and predicts nanofibril alignment during hydrodynamic assembly.^21−23^ The digital twin gives access to the complete local 3D-orientation distributions and serves as a numerical “microscope” that reveals aspects of the particle behavior with implications for rheology, mixing, and assembly in systems with anisotropic nanoparticles.

**Figure 1:**
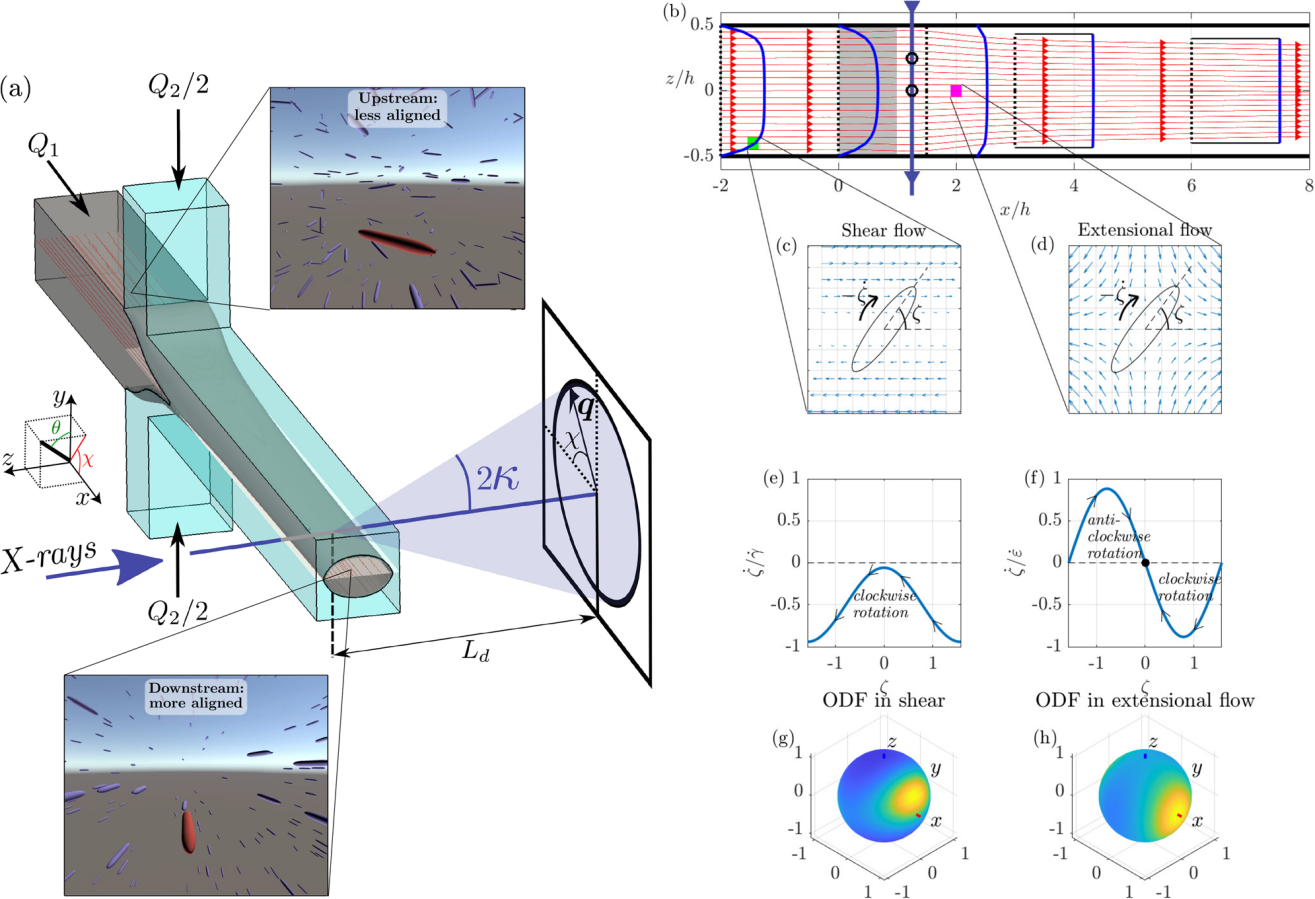
(a) Illustration of the reference flow geometry (single flow focusing, SFF), artist impressions of the upstream and downstream orientational states, and SAXS setup used to quantify the alignment. (b) Flow in the centerplane that the X-ray beam crosses: streamlines (red), velocity profiles (baseline dashed black, profile blue). The region where the sheath flows enter is shaded (the sheath flows are orthogonal to the plane shown). An X-ray beam is illustrated with a blue-gray line at *x* = 1.25*h*. The local and projected ODFs in Figure 2a–e originate from this position. (c,d) The local flow field *as experienced by a particle following the flow* at the positions marked in (b) is illustrated. The flow fields are shear flow close to the wall in (c) and extensional flow where the streamlines are compressed vertically in (d). A particle and its orientation angle ζ is defined. (e, f) Rotational velocity ζ̇ scaled with the shear (γ̇) or rate of extension (ε̇) of a nonspherical particle in shear and extension as a function of orientation ζ. (g, h) Simulated ODFs from the digital twin in the shear and extension, respectively.

Consequently, our digital twin is critical for assembly, process development, scale-up, and industrialization of promising material concepts utilizing biobased and human-made nanofiber components. Such material concepts often have innovative mechanical, electrical, or biological functionalities,^24−26^ and in the particular case of cellulose, alignment of CNF was recently identified as a key enabler for high-performance materials.^1^ We aim at developing an in-depth understanding of the mechanisms that enable assembly of stiff and strong filaments with highly aligned CNF; the digital twin framework is therefore used to investigate alignment in flow geometries (illustrated in Figure 1a) that have been used to align and assemble biobased nanofibrils into filaments.^23^

First the flow and its effect on fibrils are reviewed. After this, experiments and orientation simulations are compared, starting with the measures needed to extract the actually measured quantity from the simulations. With a proper comparison protocol established, five different models for *rotary diffusion* (the Brownian relaxation toward isotropy from an aligned state) with between one and five parameters are calibrated and compared. Eventually, a preferred model with three parameters is identified as the best. This model is then used to investigate the complete spatially resolved orientational states in complex flow situations.

## Results and Discussion

### Flow, Orientation Measurements, and Simplistic Particle Behavior

This study is based on flow focusing as illustrated in Figure 1a: a high-viscosity CNF dispersion (*Q*_1_, gray) is shaped into a thread by low-viscosity outer (or sheath) flows (*Q*2, blue).^27^ If this flow system is used together with a pH controlled dispersion-gel transition,^28^ gel threads with aligned fibrils can be created. After drying, the threads form continuous filaments with attractive mechanical properties.^23,29^ It has been demonstrated in a previous work^18^ that Computational Fluid Dynamics (CFD) can reproduce this flow at great accuracy by comparing simulated data with Optical Coherence Tomography (OCT) measurements. This agreement is illustrated in Figures S1 and S2.

The experimental data of the present work, SAXS-measurements of fibril alignment, are obtained by placing the flow setup in an intensive X-ray beam from a synchrotron and obtaining scattering images as shown in Figure 1a. Measurements at different streamwise positions are obtained by traversing the flow channel. The X-ray scattering is the result of the alignment (and size) of nanoparticles that the photons pass while traversing the flow channel. From the scattering images, the projected orientation distribution of nanoparticles in the flow is obtained as the normalized intensity variation at a given radius (*q*-value) as explained in Figure S3. The resulting orientation distributions are the integrated result of particle rotation driven by the flow and rotary diffusion experienced by the particles as they are convected along streamlines. Prediction of particle alignment therefore necessitates understanding how the flow at different positions affects particles.

Figure 1b–h is a comprehensive summary of the flow situation in the region spanned by the beam, including an introduction to the coupling between the flow and particle rotation.

First, streamlines and velocity profiles in the plane defined by the main flow direction and the beam are shown in Figure 1b. The channel walls are black, calculated streamlines are red, the velocity profiles of the high-viscosity core fluid at selected positions are blue, and an example X-ray beam is shown as a vertical line with downward arrows. The shaded region shows where the sheath flows enter.

The blue velocity profiles in Figure 1b show that the flow of the core fluid develops from a parabolic-like channel flow at *x*/*h* < 0 to a plug flow with a flat velocity profile for *x*/*h* > 2, where the core fluid is surrounded by low-viscosity sheath fluid. Further details of the flow can be deduced in Figures S1 and S2.

When a nanofiber is convected by the flow, its alignment will be affected by the local flow velocity variation. Such variations *as seen by a particle convected by the flow* at the two positions marked with green and magenta squares in Figure 1b are illustrated in Figure 1c,d, respectively. Close to the solid wall at the green square, there is a shear flow (Figure 1c), and further downstream, where the thread has formed and is stretched, the flow is extensional (position marked by a magenta square in Figure 1b and extensional flow illustrated in Figure 1d).

The flow fields experienced by particles following the flow might require some additional explanations. They are obtained by a Galilean transformation from a frame of reference fixed in the laboratory to a frame of reference following a particle. In the shear flow close to the lower wall, shown in Figure 1c, the reference particle in the center will pass particles below it (that have a lower absolute velocity). From the perspective of the reference particle, the particles below will therefore move backward and vice versa for particles above. A similar reasoning can be made to understand the extensional flow (Figure 1d), where the acceleration causes the distance between the reference particle and particles both in front of and behind it to increase. Consequently, in extensional flow particles behind the reference particle seem to move backward, as indicated by the arrows in Figure 1d.

Both shear and extension cause alignment in the mean if the rate of deformation is high enough to overcome diffusion (i.e., the Peclet number *Pe* has to be large enough). However, the nature of the particle rotation, which is given by eqs 5 and 6 in the Methods section, is different for the two cases, as illustrated in Figure 1e,f and explained in the following. In the shear of Figure 1c, the particle rotates clockwise; i.e., the rotational velocity ζ̇ is negative for all ζ, as seen in Figure 1e. The particle rotates slower, and thus spends more time, at the positions ζ = 0 and π, but it never ceases to rotate. This motion is often called tumbling or flipping.

In the extensional flow of Figure 1d, the situation is different (see Figure 1f): the particle does not tumble but instead rotates toward the position ζ = 0, which is a fixpoint since the rotational velocity ζ̇ = 0 for this orientation. For nonstiff particles, there is an additional and very important difference between shear and extensional flow. In shear, long, elastic particles undergo transitions into buckling and twisting motions,^30^ whereas an extensional flow always stretches and aligns particles.

The difference in rotation of single particles in shear and extension causes different collective behavior,^19,31^ and the behavior of individual particles will be different at high and low *Pe* and different concentrations.^32,33^

The focus here is on the collective behavior of particles. Statistically, the collective alignment state of the nanofibrils is characterized by the orientation distribution function (ODF), Ψ. This function exists on a unit sphere and the simulated distributions at the green and magenta squares in Figure 1b are shown in Figure 1g,h, respectively. The orientation distribution function has a high value (yellow on the spheres) at positions on the sphere that corresponds to directions in which many particles are oriented. In the shear at the green square, the combined effects of rotation driven by the shear and rotary diffusion leads to that the most probable alignment is inclined with respect to the flow direction, whereas the nanofibrils tend to align in the flow direction in the extensional (accelerating) flow at the magenta triangle.

### Comparing Measurements and Orientation Simulations

**Figure 2:**
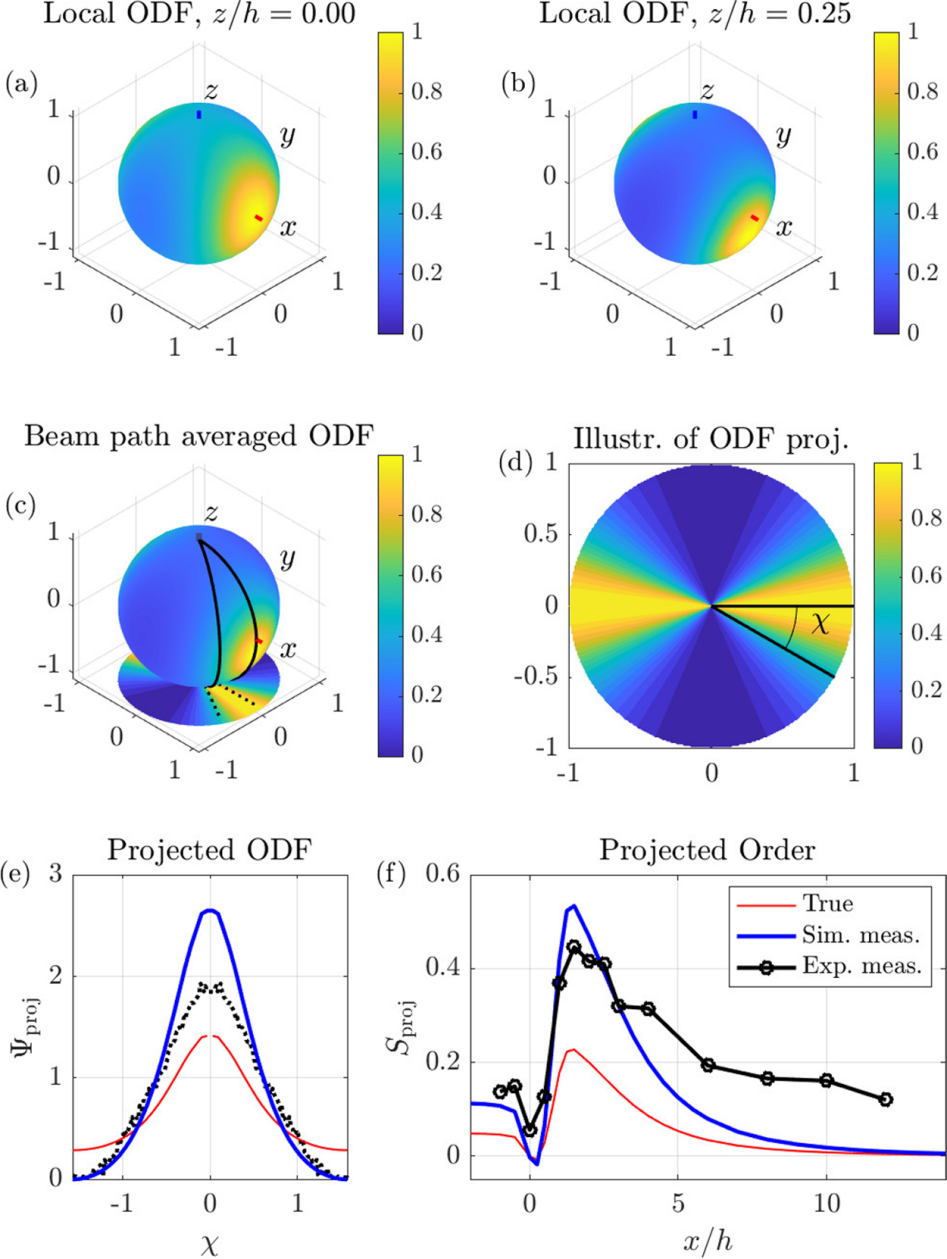
(a, b) Local orientation distribution functions at the positions marked with circles in Figure 1b: (*x*, *y*) = (1.5*h*, 0) and *z*/*h* given above each panel. (c) Average orientation distribution along the beam path marked with a gray vertical line in Figure 1b. The projection in the *xy*-plane generated from simulation data is also shown. (d) Illustration of the projection of the beam-averaged ODF and definition of the angle χ. (e) Projected orientation distributions. The true distribution is shown in red. The blue curve shows the renormalized projected ODF with the minimum set to zero (the latter is denoted the measured ODF). (f) Order *S*_proj_ of the projected ODF along the centerline: experimental and simulated measurements together with the true order given by the simulations. The C1 model with the parameter values in Table 3 was used. All ODFs are normalized with their maximum value. Animated data are available in movie M1.

Note that in order to describe the alignment state of the nanofibrils in the channel, one ODF sphere at every point in the channel is necessary. Mathematically, the complete ODF is therefore a function of not less than five parameters, Ψ(*x*, *y*, *z*, ϕ, θ), where ϕ and θ are spherical coordinates on the sphere as defined in Figure 1b. If the flow field is known, Ψ can be calculated with eqs 3–5 presented in the Methods section. Figure 2a,b shows two different local ODFs, taken at the positions marked with circles in Figure 1b. Thus, the X-ray beam passes positions with different ODFs and the total scattering is in fact a footprint of the *average* orientation distribution along the beam.

The averaged ODF can be obtained from the simulations and the average ODF along the beam at *x*/*h* = 1.5 is shown in Figure 2c. A simulated projected orientation distribution is in turn obtained from the averaged ODF by integration of wedges as illustrated in Figure 2c. A graphical representation of the projected ODF is shown in Figure 2d and in the *xy*-plane of (c). The resulting one-dimensional ODF is shown in Figure 2e where the red curve shows the projected ODF.

The experimental data include an isotropic scattering of unknown amplitude from nonorientable particles and agglomerates.^19^ Therefore, the postprocessing of the experimental data includes setting the minimum value of the projected ODF to zero at the streamwise position of maximum alignment. If the same procedure is applied to the projected ODF extracted from simulated data, we obtain what we call the *simulated measurements* since it is simulated data that has been postprocessed in the same manner as the experimental data. The simulated measurements are shown in blue in Figure 2e.

Thus, it is the blue curve with simulated measurements in Figure 2e that should be compared to the experimental data shown with black dots, while the red curve with true simulated order represents the projection of the *actual* orientation state.

The projected order parameter *S*_proj_ is calculated from Ψ_proj_ according to eq 9 in the Methods section. The value of *S*_proj_ ranges from −0.5, when the projections of all particles are aligned in the *y*-direction, via 0, for an isotropic situation, to 1 when the projections of all particles are aligned in the *x*-direction.

We are now ready to compare experiments with simulations and calibrate our alignment models. Figure 2f shows the streamwise development of the experimentally measured order parameters in black together with simulation results: simulated measurements (blue) and true order (red). Here, the most simplistic rotary diffusion model, the C1 model, has been used (see Tables 1 and 2 and the Methods section for explanations and definitions of the different models). Comparing the blue and black curves, it is seen that the C1 model captures the general behavior of the projected order well: the order is constant for *x*/*h* < 0.5 followed by a slight decrease at the beginning of the focusing and a rapid increase of *S*_proj_ during the acceleration phase of the focusing, where the extensional flow leads to particle alignment. A maximum in *S*_proj_ occurs around *x*/*h* = 1.5. Downstream of the maximum, the streamlines are straight (cf. Figure 1a) and the flow is constant. Thus, the velocity gradients are very small and the particle behavior is dominated by rotary diffusion, which decreases the alignment as the particles are convected downstream.

**Table 1:** Physical Mechanisms Included in the Different Rotary Diffusion Models^a^

	model				
	C1	V1	FT1	C2	FT2
orientation dependent mobility	–	×	–	–	–
hydrodynamic interactions	–	–	×	–	×
multiple lengths of the nanofibrils	–	–	–	×	×

a The letters in the model: C, constant; V, varying (with orientation); FT: Folgar–Tucker. The numbers 1 and 2 indicate that the models contain one or two length fractions, respectively. The C2 model is found to be appropriate for the CNF case.

**Table 2:** Expressions for the Rotary Diffusion, *D*_r_, and Other Information for the Five Models^a^

model	fractions	expression for *D*_r_	no. of params	params modeled
C1	1	*D*_r_^C^	1	*D*_r_^C^
V1	1	*D*_r_^V^	1	*D*_r_^C^
FT1	1	*D*_r_^C^ + *D*_r_^FT^	2	*D*_r_^C^, *C*_*i*_
C2	2	*D*_r_^C^	3	*D*_r,fast/slow_^C^, α_fast_
FT2	2	*D*_r_^C^ + *D*_r_^FT^	5	*D*_r,fast/slow_^C^, *C*_*i*,fast/slow_, α_fast_

a Exact definitions of the parameters are found in eqs 11–14.

Even though the overall behaviors of the black and blue curves in Figure 2f are similar, they differ in the details and it will now be shown that an appropriate model for the rotary diffusion is able to give a near perfect reproduction of the experimentally obtained order parameter at all positions.

In some cases it might be possible to create a highly aligned experimental reference. In such cases, it is not necessary to distinguish between the simulated measurements and true data^20^ since a highly aligned system does not contain an isotropic part to be subtracted. However, the approach used here, where simulated and experimental measurements are compared before the true alignment state is deduced from the simulations, is necessary if no highly aligned reference is available.

### Selection of an Appropriate Rotary Diffusion Model

Five different rotary diffusion models, which are intended to model different combinations of physical mechanisms as indicated in Table 1, are evaluated. The explicit expression of *D*_r_ for each model is provided in Table 2, but complete definitions are left for the Methods section. These models will now be evaluated and a preferred model will be chosen.

#### One-Fraction Models

**Figure 3:**
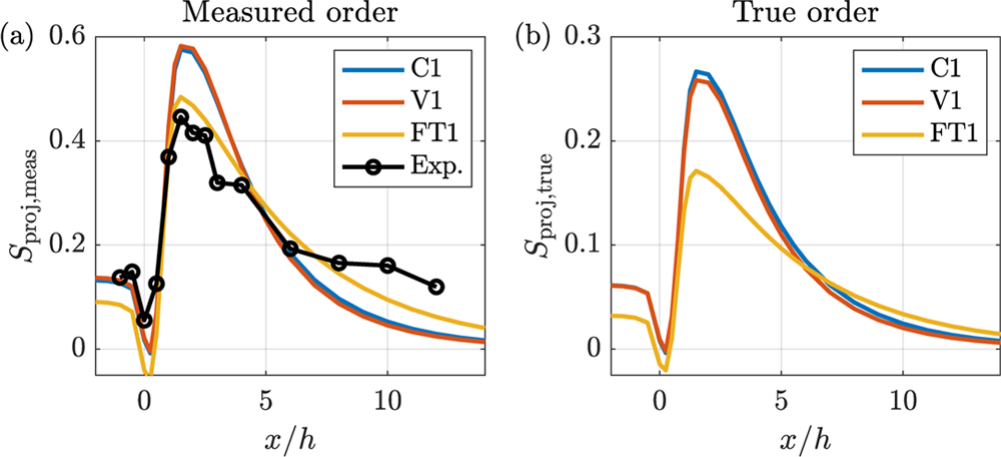
Order parameter of the beam averaged and projected ODF (i.e., *S*_proj_) along the centerline for the single-fraction models as indicated in the legend. (a) Experimental data compared to the simulated measurements. (b) True order. The models and parameters are specified in Tables 2 and 3, respectively.

Figure 3 shows the order parameter along the centerline for the single-fraction models C1, V1, and FT1. For each model, the parameters are varied so that the sum of the squares of the difference between the experimental and simulated curves in Figure 3a is minimized. The resulting parameters, together with the minimum error for each model, are given in Table 3.

**Table 3:** Optimal Parameter Values for the Different Models^a^

	*D*_r_^C^ [s^–1^]		*C*_*i*_			
model	fast	slow	fast	slow	α_fast_	error
C1	1.24				1	0.111
V1	1.74				1	0.123
FT1	0.75		0.28		1	0.062
C2	3.50	0.30			0.81	0.013
FT2	3.47	0.29	0.005	0.008	0.80	0.013

a The error is calculated as the sum the square of the differences between the experimental and simulated values of the order of the projected ODF for −5 ≤ *x*/*h* ≤ 15.

First, the typical level of *D*_r_ must be commented on. For a monodisperse dispersion of rigid rods, the rotary diffusion is expected to be *D*_r_ = 1/(6τ), where τ is the critical time scale at which shear thinning sets in.^13^ In the present case, τ = 16 s so *D*_r_ ≈ 0.01 s^–1^ would be expected (see the Methods around eq 1 and Figure S4). However, the values in Table 3 are approximately 100 times higher; i.e., the system relaxes to isotropy 100 times faster than what would be expected from the shear rheology. This demonstrates that the shear rheology only captures the slowest time scales in the system, whereas the present fitting to simulated measurements captures the (much faster) time scales that must be controlled if an aligned nanostructure is to be assembled.

Going into detail, it is clear in Figure 3a that the orientation dependent rotary diffusion of the V1 model does not improve the results substantially compared to the simplistic C1 model results. The FT1 model, which intends to model hydrodynamic interactions by adding a rate-of-deformation dependent component of the rotary diffusion, does a slightly better job of predicting the streamwise development of the experimental order parameter. However, significant deviations also remain for FT1 since the order at *x*/*h* < 0.5 is underpredicted, the maximum is slightly overpredicted, and the decay of the order after the maximum is considerably more rapid than in the experiments.

It is also worth noting that even though the measured orders (i.e., the order of the projected ODF calculated after subtraction of the minimum value and renormalization) from the experiments and simulations are similar, the true order including the isotropic part, shown in Figure 3b, is much lower for the FT1 model than for the C1 and V1 models. This stems from the fact that the measured orders can be similar although the underlying true order varies significantly. The digital twin is necessary to resolve this issue, since the digital twin can be calibrated against the experimental data and be used to investigate actual conditions.

#### Two-Fraction Models

In order to simulate the experimental order parameter better, more complex models must be applied. One reason behind such complexity is the length variation of the nanofibers and their interactions. Due to these aspects, the diffusive behavior of the fibrils cannot be described by a single rotary diffusion.^35^ Instead, a distribution is needed since short fibrils have a high rotary diffusion and long fibrils have a low rotary diffusion. An attempt to, to some extent, include this is done with the two-fraction models C2 and FT2. The optimal values of the high and low diffusion coefficients for these models (see Table 3) are in reasonable agreement with recent studies of CNF dealignment in flow-stop experiments.^19,36^

As a matter of fact, rotary diffusion of elongated particles in nondilute and/or complex situations always relies on fitting of multiple parameters to experimental data or detailed particle-level simulations.^13,37−39^ In this context, the present approach of extracting the actually measured quantity from the simulations makes it possible to perform this fitting from spatially averaged and projected experimental data. Estimation of the rotary diffusion from first-principles for semidilute dispersions of nonstiff elongated particles such as CNF cannot be done due to the complexity of the phenomena (see the Methods section for further discussion).

**Figure 4:**
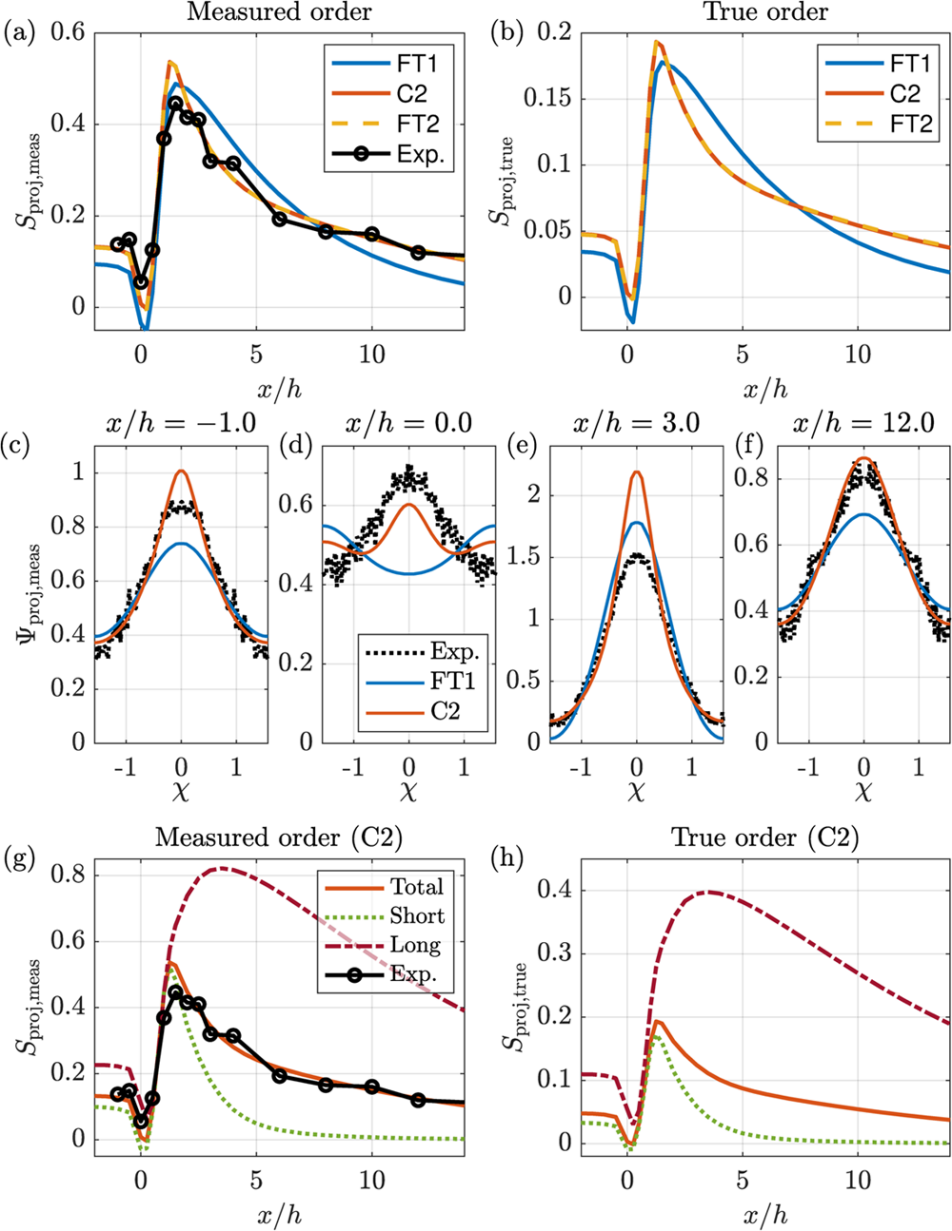
Orientation results from the double-fraction and the best single-fraction models. (a) Measured order along the centerline (experimental and simulated data). (b) True order along the centerline (only available from the simulations). (c)–(f) Projected orientation distributions at selected streamwise positions. (g, h) Measured and true, respectively, order of the projected orientation distribution for the long and short fractions. The models and parameters are specified in Tables 2 and 3, respectively. The preferred model is chosen as C2 (details in the text).

In Figure 4a, the measured orders obtained with the two-fraction models are compared with the experimental data and the best one-fraction model (FT1). The two-fraction models are seen to give a near-perfect reproduction of the experimental data. It is also clear that the shear augmented rotary diffusion of FT2 does not improve the results compared to the results of the more straightforward C2 model. The good agreement between experimental and predicted values are further elucidated in Figure 4c–f, where the projected orientation distributions are compared.

On the basis of the observations above, the C2 model is chosen as the best model for simulations of the experimentally observed alignment dynamics in the channel. A few aspects of this model will be highlighted before using it to investigate the orientational state in the channel in more detail. First, Figure 4b shows that the actual maximum of the projected order is around 0.2, even if the measured value (see Figure 4a) is around 0.45. Furthermore, the difference between the long and short fractions can be deduced from Figure 4g,h for the measured and true projected order parameter, respectively. The short (fast) fraction reaches a lower maximum order around *x*/*h* = 1.5 and decays quickly further downstream, whereas the long (slow) fraction reaches a much higher order at *x*/*h* = 3. The high order of the slow fraction remains far downstream. The digital twin also reveals that the maximum of the actual order is approximately half the value given by the SAXS data (0.2 vs 0.4).

### Complete Orientation Results in Two Flows Used for Assembly

**Figure 5:**
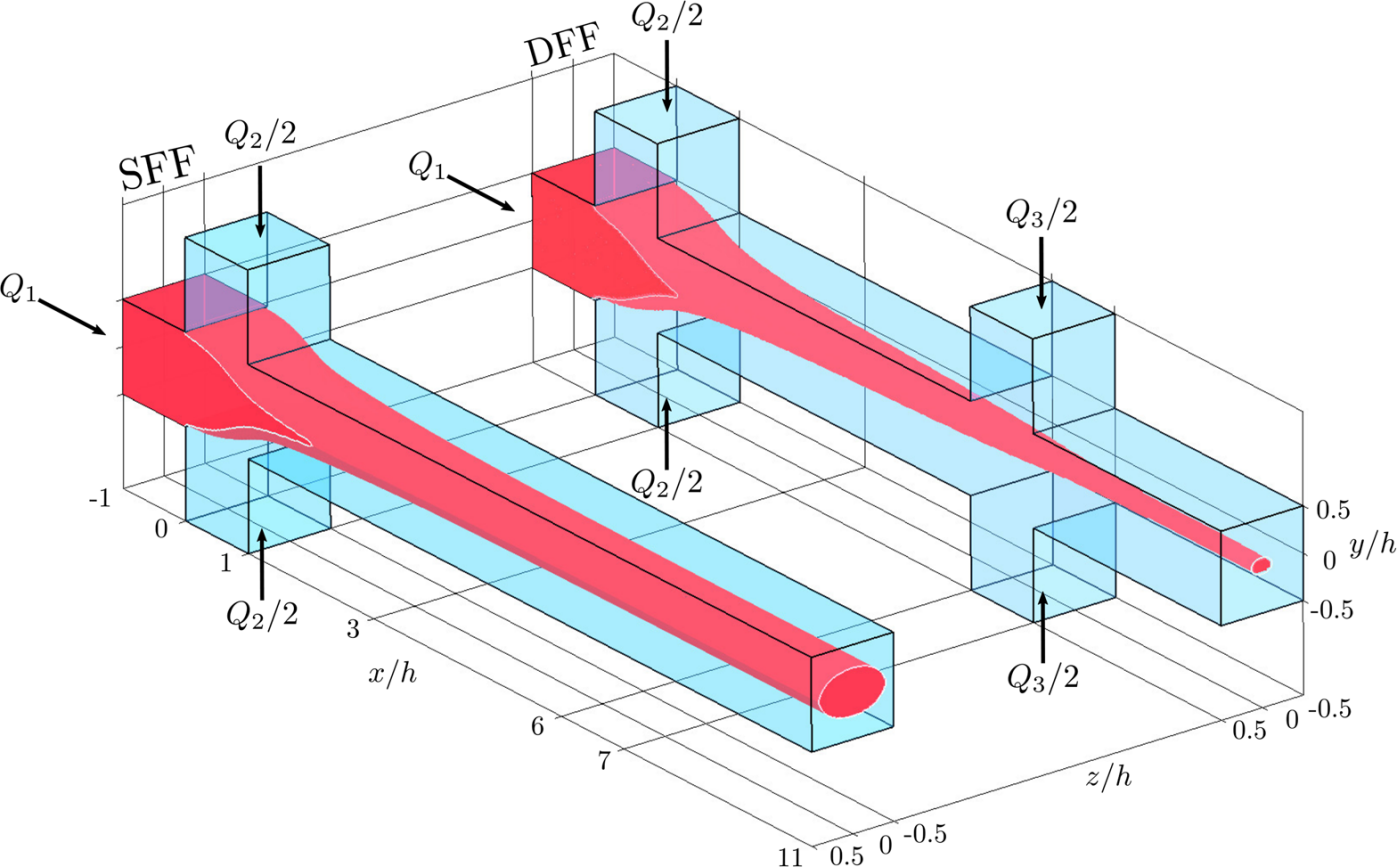
Single flow focusing (SFF) and double flow focusing (DFF) channel geometries. Red denotes the core fluid (the nanofibril dispersion) entering the central inlet channel with flow rate *Q*_1_ and forming a thread further downstream. Light blue represents the sheath fluid entering from the side channels with flow rates of *Q*_2_/2 and *Q*_3_/2, as indicated. The region occupied by the core flow is obtained from the underlying CFD model.^18^

The digital twin provided by the C2 model will now be used to investigate the orientational state in the hitherto discussed reference single flow focusing, and the full value of the digital twin becomes apparent when the results are compared with the alignment in the second generation double flow focusing (DFF) geometry (see Figure 5). In both geometries, the flow rates are chosen to match those used to assemble high-performance CNF filaments^23,29^ and PNF filaments.^40^ The DFF geometry was initially introduced to solve clogging issues (see the Methods section for more details). Below, the digital twin reveals that there are additional benefits with the DFF when used for assembly.

**Figure 6:**
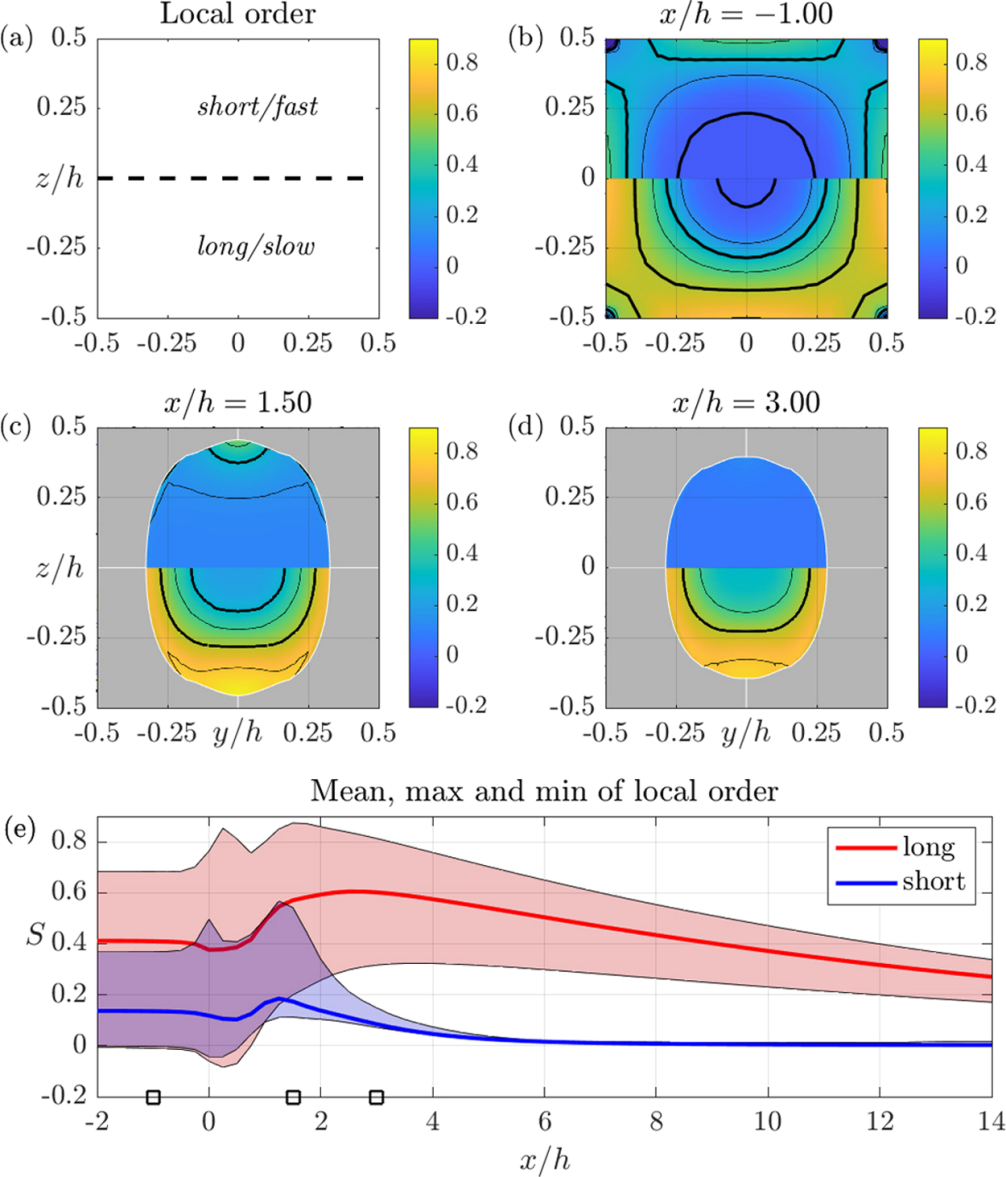
Local order *S*_local_, eq 7, in the SFF geometry (see Figure 5) obtained with the C2 model. (a) Explanation of the following panels: data for the short fraction are shown in the upper half and data for the long fraction in the lower half. (b)–(d) Cross section contours of *S*_local_ at the streamwise positions (*x*/*h*) indicated above the panels. (e) Streamwise development of mean, maximum, and minimum order over the whole cross section. The range of orders is indicated with blue and red tints for the short and long fractions, respectively. The *x*/*h* positions of the cross sections in (b)–(d) are indicated with squares on the horizontal axis.

**Figure 7:**
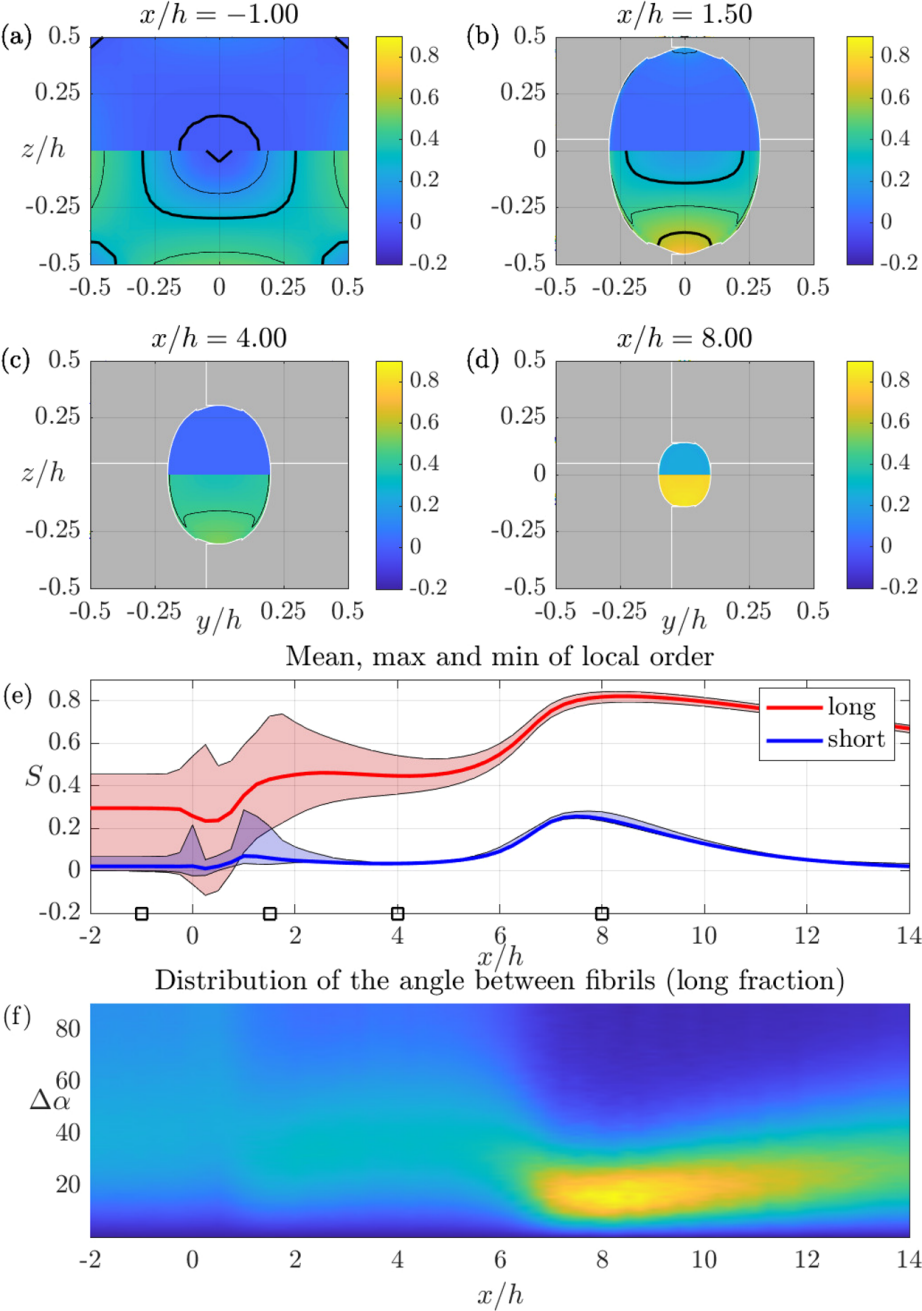
Local order *S*_local_, eq 7, and relative orientations in the DFF geometry (see Figure 5) obtained with the C2 model. (a)–(d) Cross section contours of *S*_local_ at the streamwise position indicated above each panel. (e) Streamwise development of mean, maximum, and minimum order over the whole cross section. The range of orders is indicated with a blue and red tint for the short and long fraction, respectively. The *x*/*h* positions of the cross sections in (a)–(d) are indicated with squares on the horizontal axis. (f) Distribution of the angle between fibrils as a function of streamwise position. Blue is low, and yellow is high; the scale is arbitrary.

Detailed alignment results in SFF and DFF are shown in Figures 6 and 7, respectively. In both figures, the panels a–d show cross section distributions of *S*_local_ defined in eq 7, which is 0 for an isotropic ODF and 1 if the ODF is zero for all orientations except for the *x*-direction; i.e., all fibrils are aligned. The top half of each panel shows the local order for the short (fast) fraction and the bottom half for the long (slow) fraction. Finally, the e panels show the mean (thick line), maximum, and minimum values of the local order for both fractions as a function of streamwise position. Thus, the latter graphs show the variation of order both within and between the two fractions, which are used as a first model for the real, continuous, length distribution.

Starting with the SFF geometry shown in Figure 6, it is seen that the order varies considerably over the cross sections in (b)–(d). For the long fraction, the footprint of the strong shear close to the walls at *x*/*h* < 0 in (b) remains at *x*/*h* = 3 in (d), in particular for the long fraction (bottom half of the cross sections). Consequently, the local order of both fractions in (e) shows a significant variation around the mean value at all positions (unless the order is zero; i.e., the ODF is isotropic). The higher order originates from the strong shear close to the walls of the inlet channel and remains around the rim of the thread after detachment from the walls.

The situation is quite different for the DFF geometry in Figure 7. In this case, the core flow rate is lower and the total sheath flow rate is higher than that of the SFF (see the Methods section), which is why the final size of the core is smaller, as seen in Figure 5. The differences in flow rates also imply that the shear at the wall for *x*/*h* < 0 is lower and the acceleration of the core during focusing is higher. This has several implications for the alignment. The order is lower close to the walls at *x*/*h* < 0 (cf. Figures 6b and 7a). The thread is then formed by the two sheath flows and the maximum order is obtained around *x*/*h* = 7. Figure 7 panels d and e show that the order of both fractions is high with minimal variation within each fraction after the second sheath flow. It should be mentioned that high and homogeneous fibril alignment (in dispersion) might be possible to achieve also in the SFF if the sheath flow is increased and core flow is decreased. However, we have not succeeded to prepare continuous filaments using SFF under such flow conditions.

Finally, an example of insights accessible from the complete simulations are shown in Figure 7f where the distributions of the angle between fibrils (see methods for details) at different positions is shown. This property is important for assembly, since it determines the conditions under which fibrils will interact. The distributions are relatively broad over the possible range 0–90°, except for the region where the alignment is very high (7 < *x*/*h* < 10), where a distinct peak around 20° relative alignment is found.

## Conclusions

In situ small-angle X-ray scattering and simulations have been combined to produce a digital twin that provides the full 3D orientation state of colloidal nanofibers in complex flow geometries. To start with, the results show under which conditions, and at which positions, the shorter and longer fibrils can be expected to be aligned. The variation of nanostructure over the cross section of the flow channel is revealed in terms of the alignment variation both *within* and *between* the fractions of short and long fibrils. The results also show to what extent the flow must be accelerated in order to establish a homogeneous alignment of each fraction.

In addition to the direct conclusions regarding nanostructure during assembly, three additional conclusions deserve to be mentioned. First, the alignment dynamics of the multidisperse, entangled CNFs are predicted very well by a two-fraction dispersion model assuming dilute straight rods for the particle rotation. However, the rotary diffusions relevant in the flows used for assembly are 30–300 times faster than the rotary diffusion detected by shear rheology, emphasizing the need to characterize the rotary dynamics in process-like situations. Furthermore, the coupling between the dispersion properties (length distribution of the CNFs, concentration, etc.) and the two values of rotary diffusions is yet to be understood.

Second, the complete orientational distribution functions give access to previously hidden properties such as the distribution of relative orientations. These distributions are critical to understand how particles agglomerate into larger structures, e.g., using in silico molecular dynamics simulations.

Finally, the digital twin can be used together with topology and/or geometry optimization to design flow geometries that tailor the nanostructure together with the cross sectional shape of the thread. This will lead to materials with optimal mechanical properties in combination with nutritional, biological, electrical, optical, or other innovative functions.

## Methods

### Fluid, Flow Geometries, and Flow Modeling

#### Cellulose Nanofibril Dispersion

The nanoscale particles studied in the present work are cellulose nanofibrils (CNF) in water. Such fibrils have been identified as an interesting starting point for biosourced nanostructured materials and are available from the biosphere.^2^ The CNF used in the present work was obtained by disintegrating pulp fibers and a thorough description of the CNF preparation is given in previous work.^19^ The length and diameter of the slender fibrils are 100–1600 and 1–6 nm, respectively. The surface charge density of the fibrils is approximately 600 μmol g^–1^.

The concentration of the fibrils is 0.3% by weight and the rheology is described by the Carreau model^18^
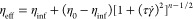
1where η_eff_ is the viscosity and the parameter values are η_inf_ = 5 mPa s, η_0_ = 1756 mPa s, τ = 16.16 s, and *n* = 0.56. As can be seen in Figure S4, the viscosity of the CNF dispersion is around 1000 times higher than that for the surrounding water at low shear rates and 20 times higher at high shear rates.

#### Single- and Double-Flow Focusing

Two flow geometries will be studied (see Figure 5), both using the concept of flow focusing.^21,22,41^ All channels have cross sections of 1 × 1 mm^2^, and the two geometries have been chosen since they can be used to assemble cellulose filaments from cellulose–nanofibril dispersions.^23,29^ In both geometries, a core fluid (red in the figures) is focused by sheath flow(s) entering from above and below. The sheath flows focus the core flow, and a fluid thread is formed in the downstream outlet channel. The two geometries differ in the number of sheath flows and are denoted single flow focusing (SFF) and double flow focusing (DFF), for the cases with sheath flows entering at one and two positions, respectively. These flow geometries have turned out to be very versatile research platforms both for assembly of filaments and for understanding of the fibril dynamics during processing.^19,23^

When SFF and DFF are used for assembly of filaments, the nanoparticle dispersion enters as the central core fluid and water with gelling agents (e.g., acid or ions) entering from the sides. The gelling agents diffuse into the core and lock the fibrils in the nanoparticle thread into a gel. If the gel thread is ejected, picked up, and dried, a filament is achieved.

The SFF geometry has found widespread use. It can be used to form threads from dispersions,^27^ prepare filaments,^22,41^ or investigate mixing and/or reaction kinetics.^42−46^ It was thus the natural choice for the initial demonstrations of the CNF assembly concept.^23^ However, when used for CNF assembly, SFF flow cannot be run for long periods of time due to clogging of gelled core fluid near the sheath inlets.

The clogging issue is solved with the DFF geometry. When used with a nongelling fluid in the first sheath flow,^47^ the assembly process can be run for long times since the water in the first sheath flow ensures that the core fluid is separated from all walls before it is reached by the gelling agent introduced in the second sheath.^48^ Due to its improved runnability, the DFF geometry has been used in more recent work for assembly of CNF, CNF/silk composites, and protein nanofibrils (PNF) as well as time-resolved mixing experiments.^29,40,49,50^

Here, the less complex SFF geometry is used in the experiments and for calibration of our numerical model of particle orientation. The resulting model is then used to extract information on the fibril behavior in the technically more relevant DFF geometry. The flow rates are the same as used when filaments are assembled:^23,29^*Q*_1_ = 23.5 mL/h, *Q*_2_ = 27 mL/h in the SFF and *Q*_1_ = 4.1 mL/h, *Q*_2_ = 4.4 mL/h, and *Q*_3_ = 24.6 mL/h in the DFF. Note that the ratio between the sheath(s) and core flow is much higher in the DFF case than in the SFF. A consequence of this is seen in Figure 5, where the downstream thread is seen to be much smaller in the DFF case than in the SFF case. This also means that the stretching of the core flow is considerably stronger in the DFF than in the SFF.

#### Numerical Modeling of the Flow

In order to model the behavior of the nanofibers in the flow, a detailed description of the flow velocity **u** = (*u*(*x*, *y*, *z*), *v*(*x*, *y*, *z*), *w*(*x*, *y*, *z*)), where (*x*, *y*, *z*) are Cartesian coordinates and (*u*, *v*, *w*) are the corresponding components of the velocity, must be at hand since the particles rotate due to spatial variations of the flow velocity.

A detailed description of the flow field can be obtained through computational fluid dynamics (CFD). The present work relies on a recently developed numerical model of the flow, which was thoroughly validated against experiments.^18^ The key elements of the model are (i) the use of measured rheological properties of the CNF dispersion and (ii) the introduction of an effective interfacial tension acting on the defacto interface between the region with a high concentration of the particles (the core) and the region with pure solvent (the sheath flows). In the present work, the effective interfacial tension σ_eff_ = 0.054 mN m^–1^ (as a comparison, the interfacial tension between water and air is ≈70 mN m^–1^ at room temperature).

The flow is modeled with the core and sheath flows (water) as two immiscible fluids in the open source CFD software OpenFOAM.^51^ This setup has been demonstrated to predict the flow with great accuracy and detail,^18^ as demonstrated in Figures S1 and S2.

### Orientation Modeling and Measurements

#### Smoluchowski Equation

Orientation modeling with the Smoluchowski equation together with selection and calibration of models for the rotary diffusion forms the core of the present work (a graphical illustration of the relation between experiments and simulations is provided in Figure S5). The collective rotational state of nonspherical particles with one rotary symmetry can be described by an orientation distribution Ψ(*x*, *y*, *z*, ϕ, θ), where ϕ and θ are spherical coordinates. The orientation distribution Ψ is normalized so that
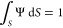
2where *S* is the surface of the unit sphere. The evolution of Ψ for monodisperse particles is given by a Smoluchowski equation:^52^
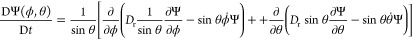
3where D/D*t* is the material derivative following the fluid, *D*_r_ is the rotary diffusion, θ̇ and ϕ̇ are the rotational velocities of a particle in the local flow field; the dot is used to denote the temporal derivative. The rotational velocities are obtained from the rate of change of the director of a particle **p**, defined as
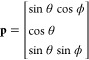
4

Assuming ellipsoidal particles and very viscous (Stokes) flow, the rate of change of **p** in the local flow field is^13,14^
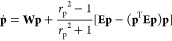
5via a projection on the unit vectors of the spherical coordinates, i.e.
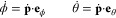
6In eq 5, **E** and **W** are the symmetric (rate of deformation) and antisymmetric (vorticity) parts of the velocity gradient tensor. The parameter *r*_p_ is the aspect ratio of the particle; *r*_p_ > 1 for prolate (cucumber-like) and *r*_p_ < 1 for oblate (pancake-like) particles, respectively. For the CNF of the present work, *r*_p_ > 25 and (*r*_p_^2^ – 1)/(*r*_p_^2^ + 1) ≈ 1.

At this point, a comment regarding eqs 5 and 6 must be made. These equations describe the rotation of stiff and straight rod-like particles. Using them as a basis to model rotation of the entangled and nonstiff CNF could be questioned, and more complicated relations between the flow gradients and particle rotation could perhaps be considered. However, the present work is restricted to the assumption that the coupling between flow and particle rotation is described by eqs eqs 5 and 6.

Equation 3 is discretized with fourth-order central differences on the domain 0 ≤ θ < π, 0 ≤ ϕ < π using the fore–aft symmetry condition Ψ(ϕ + π, θ) = Ψ(ϕ, π – θ). The resulting system is solved in time along one streamline at a time with the MATLAB function ODE15s.

#### Order Parameters

The information in the 3D ODF is reduced in two ways. The first is by calculating the local order parameter *S*_local_ as^13^
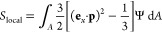
7where **e**_*x*_ is the unit vector in the streamwise direction *x* and the integration is made over the sphere.

The second data reduction is extraction of the beam averaged and projected ODF (see Figure 2). The projected ODF Ψ_proj_(χ) is normalized according to
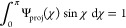
8and this orientation distribution is the simulated equivalence of the SAXS results. From Ψ_proj_, the projected order parameter *S*_proj_ is calculated as
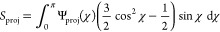
9

#### Distributions of Angles between Fibrils

The angle Δα_*kl*_ between two fibrils with orientations **p**_*k*_ and **p**_*l*_ is calculated as
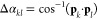
10The distribution of Δα at a certain position is obtained by generating a set of orientation vectors **p**_*i*_ that fulfill the local orientation distribution function Ψ and generate the distribution of all relative angles.

#### Rotary Diffusion Models

The key to model this physical system correctly lies in the rotary diffusion *D*_r_.^53^ Here, models based on the following three assumptions will be evaluated: (i) a constant *D*_r_^C^, (ii) an orientation state and direction dependent *D*_r_^V^,^13^ and (iii) a flow gradient dependent *D*_r_^FT^.^54,55^ The superscripts are *C* for constant, *V* for varying, and FT for Folgar–Tucker.

The starting point for modeling the rotary diffusion for rodlike particles in a semidilute suspension can be taken to be a constant scalar value *D*_r_^C^, which is related to physical parameters in the following manner:^13^
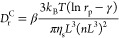
11where *k*_B_ is Boltzmann’s constant, *T* is the absolute temperature, η_s_ is the dynamic viscosity of the solvent, *L* is the particle length, and *n* is the number of particles per unit volume. The two parameters β and γ are nondimensional correction factors for particle interactions and particle shape, respectively. Here, this expression is not used explicitly, since we will be fitting the rotary diffusion to experimental data.

If the orientation distribution deviates from an isotropic distribution, the rotary diffusion in eq 3 can be expected to vary with **p** (or, equivalently, ϕ and θ) since aligned particles have more freedom to move than isotropic ones:^13^
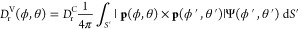
12where *S* is the surface of the unit sphere.

The effect of hydrodynamical interactions on the rotary diffusion can be modeled as^53,54^
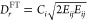
13where *C*_*i*_ is a constant and *E*_*ij*_ are the elements of the rate of strain tensor **E** and the summation convention is applied.

#### One- and Two-Fraction Models

It has been observed that the relaxation toward isotropy of a system with aligned CNF, such as the ones used in the present experiments, cannot be described by a single time scale (or with a single rotary diffusion).^36^ Instead, the relaxation behavior is a process with multiple time scales, which can be related to the distribution of particle lengths in the dispersion: the alignment of short particles decay fast whereas longer particles remain aligned for a longer time (cf. eq 11). Here, an attempt to incorporate the fact that the suspension is not monodisperse is made by assuming that it can be described by two independent fractions, each governed by eq 3 with different *D*_r_. The two fractions have separate orientation distributions Ψ_fast_ and Ψ_slow_ obtained with larger and lower *D*_r_, respectively. The complete orientation distribution is then obtained as
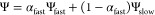
14where α_fast_ is 1 if all fibrils can be considered to belong to the fast fraction and 0 if all fibrils are slow.

Eventually, five different models will be evaluated as specified in Table 2. The parameters for each case are determined by fitting simulated data to experiments.

### Orientation Measurements: In Situ X-ray Scattering

The orientation calculations are compared with small-angle X-ray scattering measurements. The experiments were performed at beamline P03 of the Synchrotron PETRAIII at DESY, Hamburg, Germany,^56^ and are identical to previously reported measurements.^19^ A schematic of the setup is shown in Figure 1. The flow channel is mounted so that an X-ray beam measuring 26 × 22 μm and having a wavelength λ = 0.95 Å traverses the core and the photons are scattered by the material that the beam passes. The detector (Pilatus 1 M, Dectris) has a pixel size of 172 × 172 μm and is positioned at a distance of 7.5 m from the flow channel.

If the nanofibers are aligned, the scattering will be anisotropic and a projected orientation distribution of the nanofibers in the beampath can be determined as the sum number of the scattered photons for 0.25 nm^–1^ ≤ *q* ≤ 0.5 nm^–1^ for each angle χ. Some further details on this are illustrated in Figure S3. The channel is traversed so that measurements along the centerline of the channel are obtained.

There are two important aspects that must be considered when these data are compared with the calculations. The first is that we obtain the projected orientation of the mean orientation distribution along the beam path. The second is that nonorientable particles and agglomerates contribute with an isotropic part of the scattering pattern that is hard to separate from background noise unless special care is taken.^19^

## Supporting Information Available

Comparison of experimental and numerical thread shapes, comparison of the experimental and numerical velocity data, explanation of how the projected orientation distribution is extracted from the diffraction images, rheology of the fluids used, and illustration of how experimental and numerical methods are connected (PDF)
Animation of orientation distribution functions along five streamlines for the C1 model with *r*_p_ = 20 and *D*_r_ = 0.40 rad^2^/s (MOV)

## Supplementary Material

nn1c07769_si_001.pdf

nn1c07769_si_002.mov
