# Supplementary material for: Nanofibril Alignment during Assembly Revealed by an X-ray Scattering-Based Digital Twin
Source: ACS Nano. 2022 Feb 1;16(2):2120–32. doi: 10.1021/acsnano.1c07769 (PMC8867913; doi:10.1021/acsnano.1c07769)
Supplement: Supplementary file 1 — nn1c07769_si_001.pdf [file nn1c07769_si_001.pdf]

Supporting Information
Nanofibril Alignment during Assembly Revealed by an X-ray Scattering-Based Digital Twin

V. Krishne Gowda,[†,‡] Tomas Rosén,[¶,§,‖] Stephan V. Roth,[⊥,¶,§,‖] L. Daniel Söderberg,[‖,¶,§,‡] and Fredrik Lundell[*,†,‡,§]

†*Department of Engineering Mechanics, Royal Institute of Technology, 100 44 Stockholm, Sweden*

‡*FLOW, Royal Institute of Technology, 100 44 Stockholm, Sweden*

¶*Treesearch, Royal Institute of Technology, 100 44 Stockholm, Sweden*

§*Wallenberg Wood Science Center, Royal Institute of Technology, 100 44 Stockholm, Sweden*

‖*Department of Fibre and Polymer Technology, Royal Institute of Technology, 100 44 Stockholm, Sweden*

⊥*Deutches Elektronen-Synchrotron DESY, 22607 Hamburg, Germany*

E-mail: frlu@kth.se

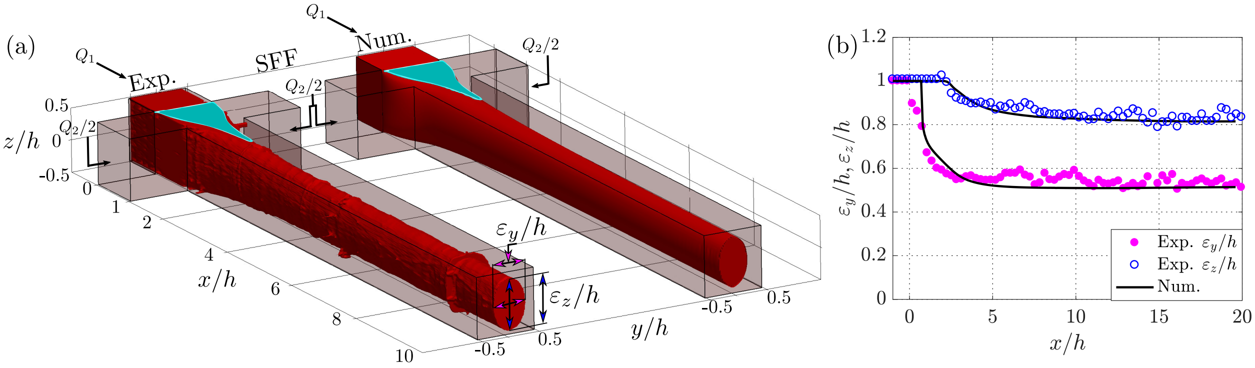

Figure 1: (a) Experimental and numerical views of 3D thread dispersion in SFF channel geometry. The cyan colour at the top plane $z/h = 0.5$ indicates the morphology of the wetted region created by the core fluid dispersion before the detachment from the top and bottom walls. (b) Evolution of thread width ε_y/h and height ε_z/h as a function of downstream positions x/h. These plots have been prepared using the data from a previously published study.[1]

Shape of the dispersion thread in the channel

Figure 1 (a) shows the region occupied by the core fluid dispersion obtained with optical coherence tomography measurements (left) and captured through numerical computations (right). The excellent agreement is underscored in Figure 1 (b), where the experimental and numerical values of the position of the vertical (ε_z/h) and horizontal (ε_y/h) edge of the thread are compared.

Velocity field comparison:

centreline and cross-sectional flow profiles

The velocity field in the SFF geometry is illustrated in Figure 2. Experimental and numerical data is shown to be in excellent agreement. In Figure 2 (a), the velocity on the centerline is shown at different streamwise positions. The centerline velocity is constant in the inlet channel ($x/h < 0$), deccelerates a little right where the sheath flows enters around $x/h = 0$. The slight decceleration is followed by an acceleration as the sheath flow (Q_2) reduces the cross section of the core flow (Q_1).

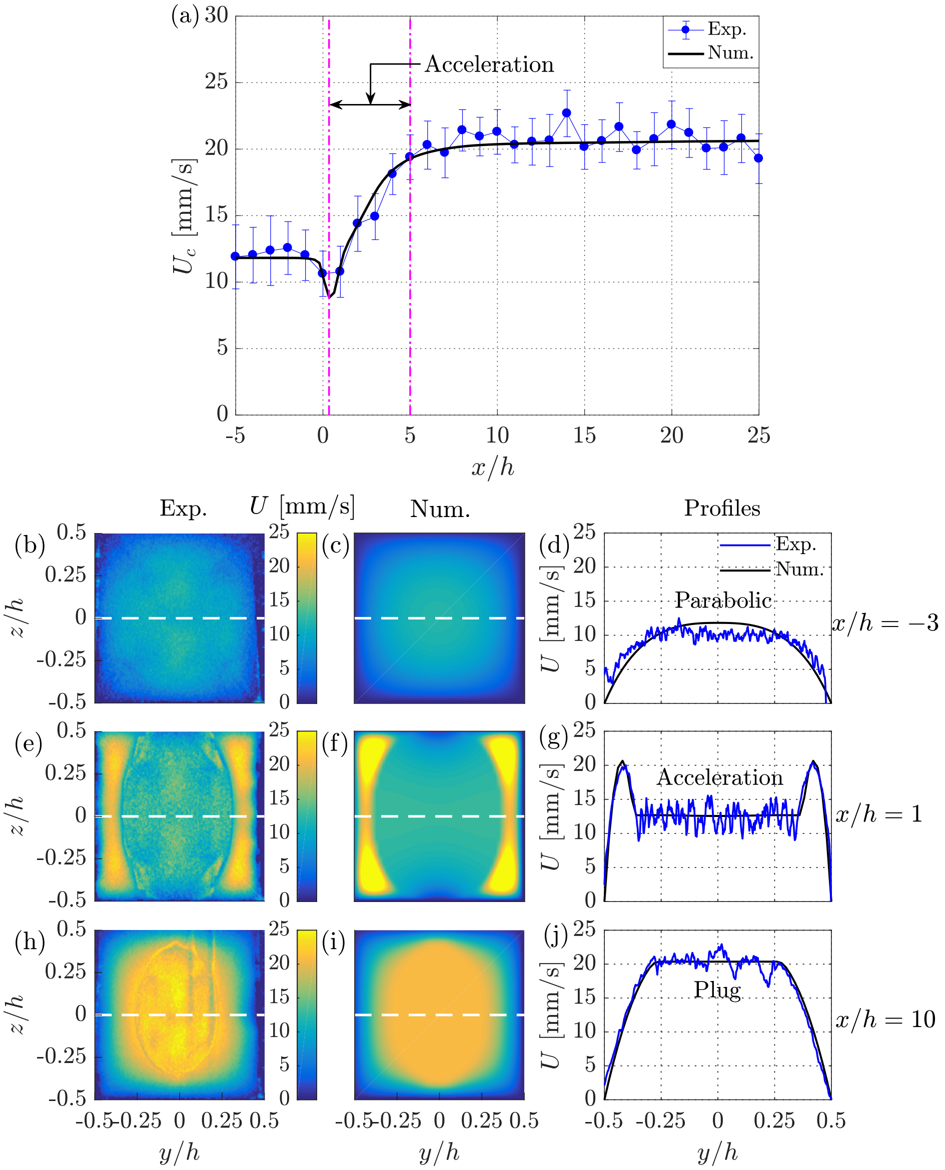

Figure 2: (a) Experimental and numerical centreline velocity U_c as a function of streamwise downstream positions x/h obtained utilising SFF channel geometry with the dispersion rheology of Figure 4. (b–j) Experimental and numerical velocity maps and flow profiles at three different locations of SFF channel geometry: $x/h = -3$ (upstream before focusing), $x/h = 1$ (end of focusing) and $x/h = 10$ (far downstream). The flow profiles in panels (d), (g) and (j) show the x-component of the velocity as a function of y/h (along the dashed white line) at $z/h = 0$. Blue line represent the experimental measurements while black solid line denote the numerical data. These plots have been prepared using the data from a previously published study.[1]

In Figure 2 (b–j), details of the cross sectional velocity distribution in the inlet channel (b–d), during the acceleration (e–g) and after the acceleration (h–j). Note that already at $x/h = 1$ in (e–g), the relatively high viscosity of the core fluid makes the velocity of the core flat, while all velocity variations appear in the low viscosity outer fluid. Also here, the numerical computation captures the experimental data very well.

Extracting projected orientation distributions from scattering images

Figure 3 shows how the 1D projected orientation distribution in (d) is extracted from the diffraction image in (a). The symmetry of the data is first used to fill intermodular gaps (b), whereafter the region in between the circles in (b) is unfolded to obtain the image in (c). From this image, the final projected (and normalized) orientation distribution shown in (d) is obtained by averaging the vertical columns (after normalization of each column according to Eq. (8)).

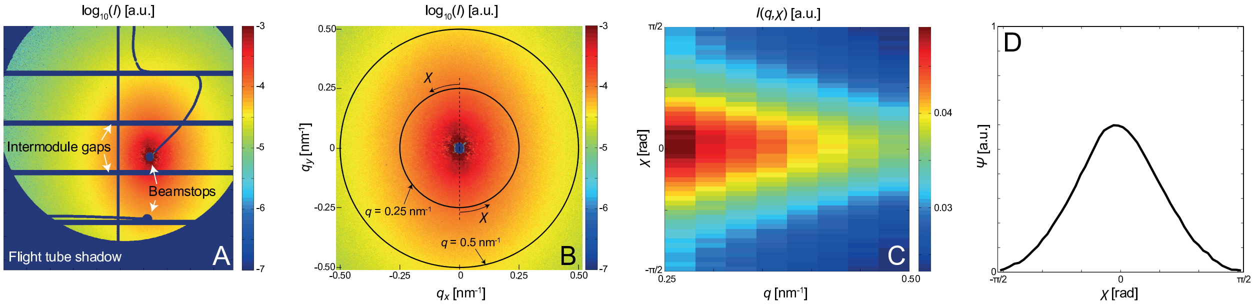

Figure 3: Obtaining projected orientation distribution from diffraction images. (a) Image after background subtraction. (b) Image with the intermodule gaps filled. The projected orientation is determined from the data in between the two circles at $q = 0.25$ nm^{-1} and $q = 0.5$ nm^{-1}. (c) The data in between the circles after unfolding and normalization. (d) Normalized projected orientation distribution after averaging of the columns in (c). The figure is a modified version of Figure 9 in the supplementary material of our previous work.[2] Reprinted with permission.

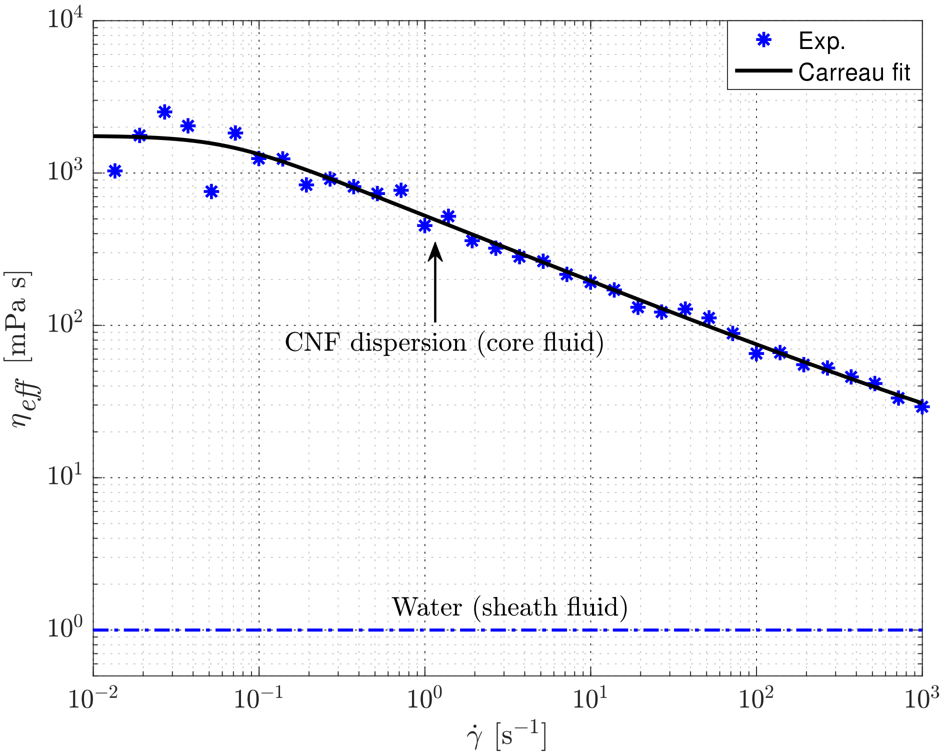

Figure 4: Shear viscosity measurements of the CNF dispersion. Experimental data are shown with blue markers, the solid black line represents the non-Newtonian Carreau model fit (Eq. (1) in the main manuscript) and the dash-dotted blue line shows the viscosity of the water in the sheath flows.

Rheology of the core and sheath fluids

Figure 4 shows the shear viscosity as a function of shear rate for the shear thinning CNF dispersion in the core flow (Q_1) and the water in the sheath flows (Q_2). Depending on the shear rate, the viscosity of the core fluid is 20 to 1000 times higher than the sheath fluid.

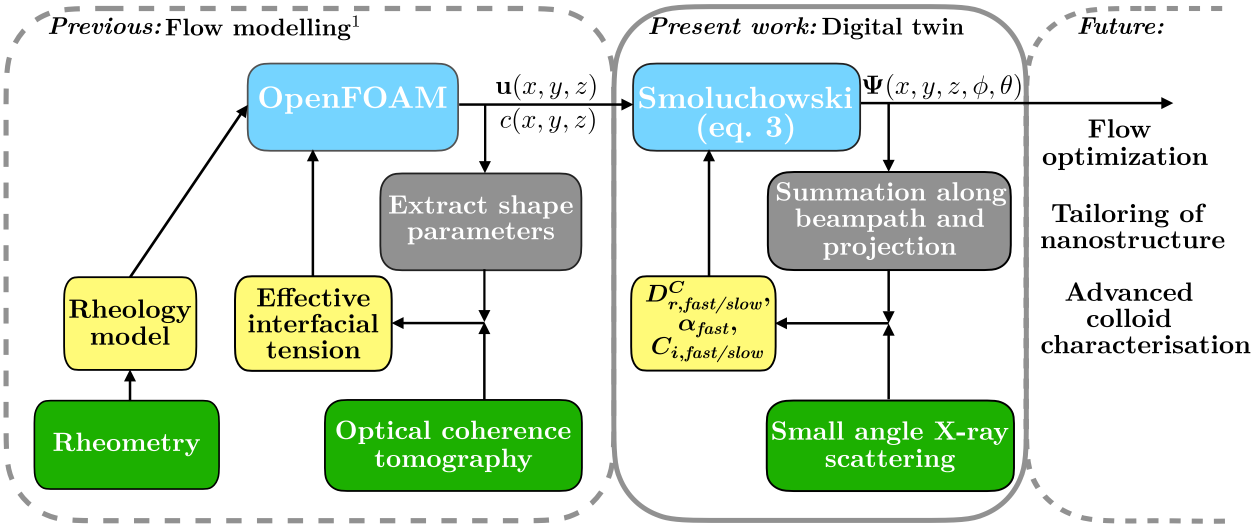

Figure 5: Schematic description of the connection between the different numerical (blue) and experimental (green) methods. The yellow boxes illustrate selection and calibration of models. The parameter $c(x, y, z)$ describes the concentration of the colloidal particles and describes the shape of the core fluid dispersion thread. The dashed block to the left shows the flow modelling[1] essential to generate the main input velocity field $\mathbf{u}(x, y, z)$, the solid central block is the present work and the right dashed block hints towards the future.

Connection between experimental and numerical methods

Figure 5 illustrates how experiments (green boxes), modelling and numerics (blue boxes) are combined in the digital twin. The flow modelling relies on rheometry, optical coherence tomography (OCT) measurements and computations in OpenFOAM, and is reported in a previous work.[1] In the present work, the detailed flow field deduced from flow modelling is used as input to the 3D Smoluchowski simulations that are matched to small angle X-ray scattering measurements.

References

1. Gowda. V, K.; Brouzet, C.; Lefranc, T.; Söderberg, L. D.; Lundell, F. Effective interfacial tension in flow-focusing of colloidal dispersions: 3-D numerical simulations and

experiments. *J. Fluid Mech.* **2019**, *876*, 1052–1076.

2. Rosén, T.; Mittal, N.; Roth, S. V.; Zhang, P.; Lundell, F.; Söderberg, L. D. Flow fields control nanostructural organization in semiflexible networks. *Soft Matter* **2020**, *16*, 5439–5449.
